# Crystal structure of 9,9-di­ethyl-9*H*-fluorene-2,4,7-tricarbaldehyde

**DOI:** 10.1107/S2056989021009464

**Published:** 2021-09-17

**Authors:** Pierre Seidel, Anke Schwarzer, Monika Mazik

**Affiliations:** a Institut für Organische Chemie, Technische Universität Bergakademie Freiberg, Leipziger Str. 29, 09599 Freiberg, Germany

**Keywords:** crystal structure, fluorene derivative, hydrogen bonding, Hirshfeld surface analysis, two-dimensional fingerprint plots

## Abstract

The title compound, C_20_H_18_O_3_, crystallizes in the space group *P*2_1_/*c* with one mol­ecule in the asymmetric unit of the cell. The fluorene skeleton is nearly planar and the crystal structure is composed of mol­ecular layers extending parallel to the (302) plane. Within a layer, one formyl oxygen atom participates in the formation of a C_arene_—H⋯O bond, which is responsible for the formation of an inversion symmetric supra­molecular motif of graph set *R*_2_^2^(10). A second oxygen atom is involved in an intra­molecular C_arene_—H⋯O hydrogen bond and is further connected with a formyl hydrogen atom of an adjacent mol­ecule. A Hirshfeld surface analysis indicated that the most important contributions to the overall surface are from H⋯H (46.9%), O⋯H (27.9%) and C⋯H (17.8%) inter­actions.

## Abstract 2

The fluorene skeleton of the title mol­ecule is nearly planar and the crystal structure is composed of mol­ecular layers extending parallel to the *302* plane. A Hirshfeld surface analysis indicated that the most important contributions to the overall surface are from H⋯H, O⋯H and C⋯H inter­actions.

## Chemical context   

Compounds featuring a fluorene moiety have been recognized as useful for a broad spectrum of applications, which range from agents for cell imaging, solar cells, organic light-emitting diodes to lasers. Furthermore, fluorene derivatives have the potential to act as artificial receptors for different ionic and neutral substrates in analogy to the known receptors possessing a benzene or biphenyl core, which, for example, are able to complex ammonium ions (Koch *et al.*, 2015 ▸; Schulze *et al.*, 2018 ▸; Chin *et al.*, 2002 ▸; Arunachalam *et al.*, 2010 ▸), ion pairs (Stapf *et al.*, 2015 ▸) or carbohydrates (Stapf *et al.*, 2020 ▸; Köhler *et al.*, 2020 ▸, 2021 ▸; Kaiser *et al.*, 2019 ▸; Lippe & Mazik, 2013 ▸, 2015 ▸; Amrhein *et al.*, 2016 ▸; Amrhein & Mazik, 2021 ▸). As a result of the manifold application possibilities of fluorenes, the syntheses of new representatives of this class of compounds are the subject of intensive research (Seidel *et al.*, 2019 ▸, 2021 ▸; Seidel & Mazik, 2020 ▸; Sicard *et al.*, 2018 ▸). Fluorene derivatives bearing halogen, formyl or amino groups are valuable starting mat­erials for a wide range of fluorene-based acyclic and macrocyclic compounds as well as polymers. Recently we have described the efficient one-step synthesis of 9,9-diethyl-9*H*-fluorene-2,4,7-tricarbaldehyde on the basis of 2,4,7-tris(bromo­meth­yl)-9,9-diethyl-9*H*-fluorene (Seidel *et al.*, 2019 ▸), which provided a threefold higher yield of the product than the previously known three-step reaction sequence (Yao & Belfield, 2005 ▸). In this work we describe the crystal structure of this fluorene derivative bearing three formyl groups.
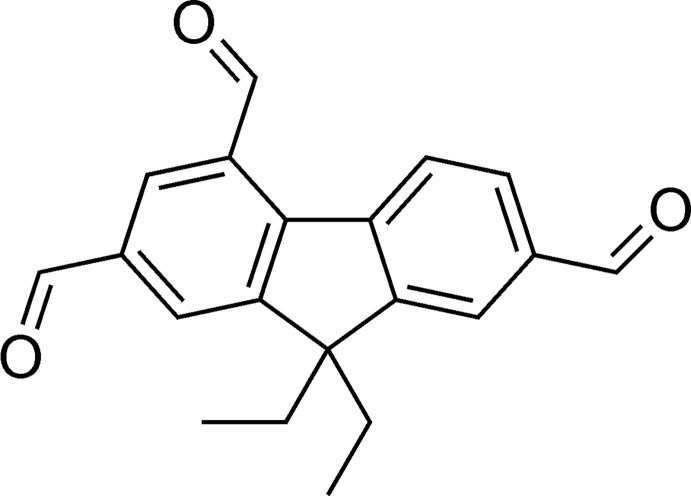


## Structural commentary   

**Figure 1:**
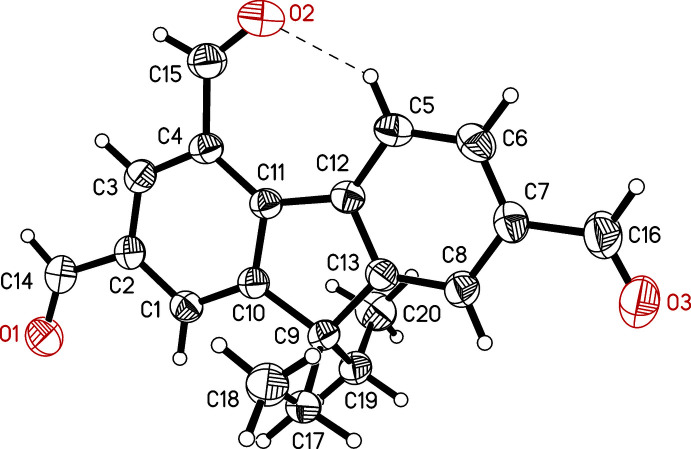
Perspective view of (**1**) including the labelling of non-hydrogen atoms. Displacement ellipsoids are drawn at the 50% probability level.

The title compound (**1**) (Fig. 1 ▸) crystallizes in the space group *P*2_1_/*c* with one mol­ecule in the asymmetric unit. The 2,4,7-substituted fluorene scaffold adopts a nearly planar geometry with the formyl groups inclined at angles of 4.2 (2), 3.5 (2) and 3.3 (2)° with respect to the fluorene moiety. These values correlate with torsion angles of −175.8 (3), −175.4 (3) and −176.7 (4)°, respectively, for the atomic sequences C3—C2—C14—O1, C3—C4—C15—O2 and C6—C7—C16—O3. The plane passing through the two ethyl groups is oriented nearly orthogonal to the plane of the fluorene unit [dihedral angle = 89.8 (1)°]. The oxygen atom O2 is involved in an intra­molecular C_arene_—H⋯O hydrogen bond [*d*(H⋯O) 2.18 Å, C—H⋯O 138°; Table 1 ▸].

## Supra­molecular features   

**Figure 2:**
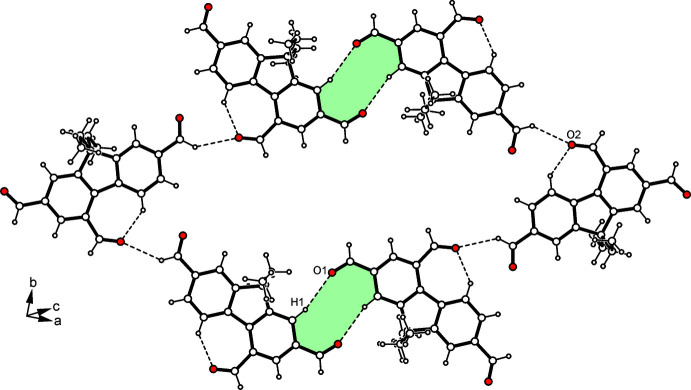
Packing excerpt of (**1**) showing selected C—H⋯O inter­actions within one layer of mol­ecules.

**Figure 3:**
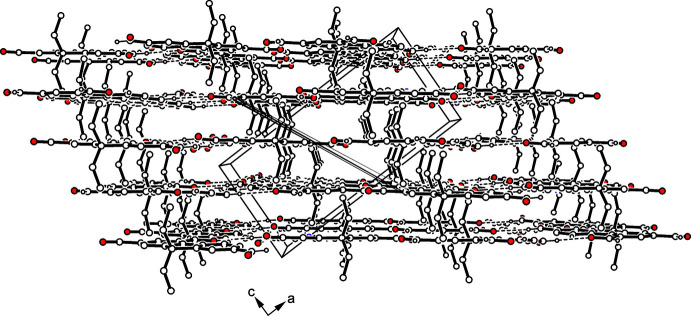
Packing excerpt of (**1**) showing adjacent layers of mol­ecules and selected C—H⋯O inter­actions within the layers. Hydrogen atoms of subunits not involved in inter­molecular hydrogen bonding are omitted for clarity.

The crystal structure of the title compound is composed of mol­ecular layers extending parallel to the (302) plane. An excerpt of the layer structure showing the mode of hydrogen bonding is depicted in Fig. 2 ▸. Within a given layer, the formyl oxygen atom O1 participates in the formation of a C_arene_—H⋯O bond [*d*(H⋯O) 2.59 Å; Table 1 ▸], thus creating an inversion-symmetric supra­molecular motif of graph-set 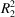
(10) (Etter *et al.*, 1990 ▸; Bernstein *et al.*, 1995 ▸; for examples of other crystal structures including such a ten-membered supra­molecular motif, see Seidel *et al.*, 2021 ▸; Stapf *et al.*, 2021 ▸). The oxygen atom O2 is connected with the formyl hydrogen H16 of an adjacent mol­ecule [*d*(H⋯O) 2.53 Å]. The steric requirements of the ethyl groups cause an offset of the mol­ecules of consecutive layers, so that neither hydrogen bonds nor π–π arene stacking inter­actions are observed in the direction of the layer normal. Consequently, the crystal appears to be stabilized by van der Waals forces in the direction of the stacking axis of the mol­ecular layers (Fig. 3 ▸).

## Database Survey   

A search in the Cambridge Structural Database (Version 5.41, November 2019; Groom *et al.*, 2016 ▸) for 9*H*-fluorene derivatives bearing a formyl group resulted in three hits, including 9*H*-fluorene carbaldehyde (SAZQIT; Dobson & Gerkin, 1998 ▸) and two ferrocene-fluorene complexes including a 2-formyl-9-fluorenyl (HAPROF) and a 2,7-diformyl-9-fluorenyl moiety (HAPRUL; Wright & Cochran, 1993 ▸). As in the case of the title compound, the 9*H*-fluorene carbaldehyde crystallized in the space group *P*2_1_/*c* with one mol­ecule in the asymmetric unit. The mol­ecular core is nearly planar and the crystal structure is characterized by the presence of C—H⋯O hydrogen bonds, which are responsible for the formation of a supra­molecular motif of graph set 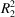
(14).

## Hirshfeld surface analysis   

**Figure 4:**
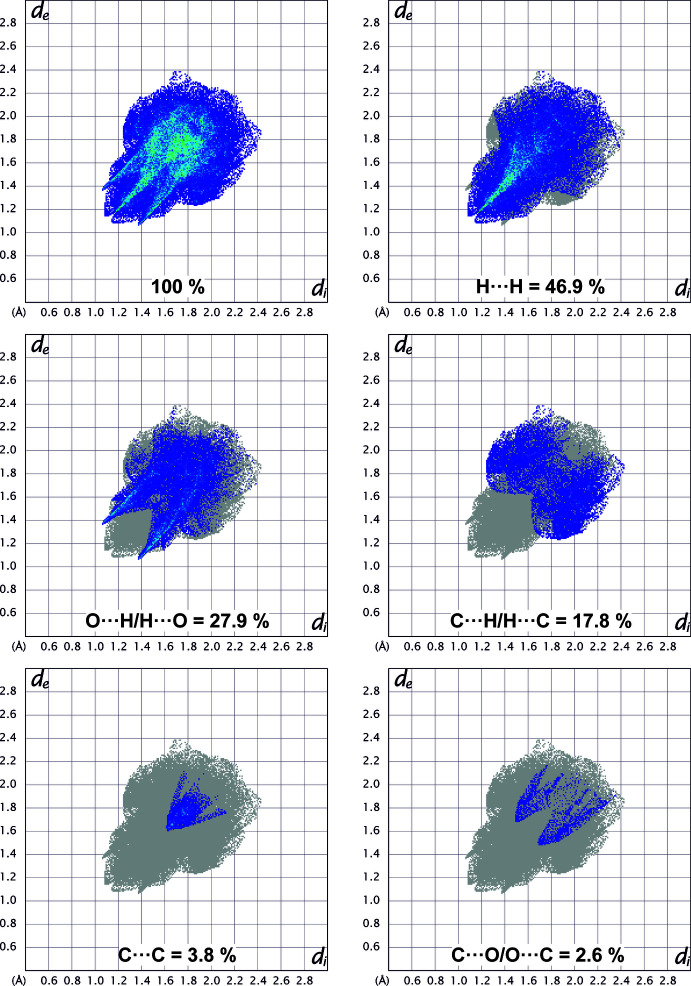
Fingerprint plot of (**1**) including the contribution of the atom⋯atom pairs to the overall surface.

**Figure 5:**
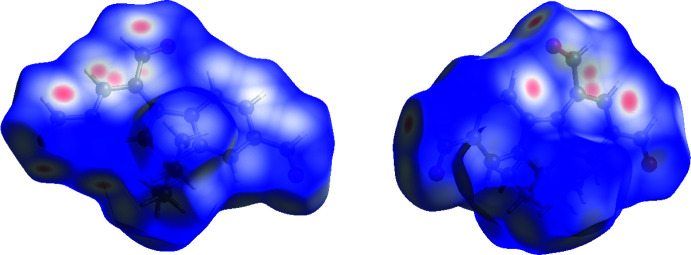
Hirshfeld surface for (**1**) mapped with *d*_norm_ (front and back views).

Hirshfeld surfaces (Spackman & Jayatilaka, 2009 ▸) were calculated and the associated 2D fingerprint plots generated using *Crystal Explorer 17.5* (Turner *et al.*, 2017 ▸). The 2D fingerprint plots (McKinnon *et al.*, 2007 ▸) are displayed within the expanded 0.4–3.0 Å range including reciprocal contacts (Fig. 4 ▸); 3D *d*_norm_ surfaces are mapped over a fixed colour scale of −0.3 a.u. (red)–1.0 a.u. (blue) (Figs. 5 ▸ and 6 ▸). The 2D fingerprint plots (see Fig. 4 ▸) indicate that the most important contributions to the overall surface are from H⋯H (46.9%), O⋯H (27.9%) and C⋯H (17.8%) inter­actions, whereas only 3.8% and 2.6% are from the C⋯C and C⋯O contacts, respectively. In addition to the fingerprint plots, the Hirshfeld plots mapped with *d*_norm_ give a hint about the significance of the close contacts. For example, the O⋯H hydrogen bonds are responsible for the intense red spots on the surface, as shown in Figs. 5 ▸ and 6 ▸.

## Synthesis and crystallization   

The title compound was prepared by an efficient one-step synthesis involving the treatment of 2,4,7-tris­(bromo­meth­yl)-9,9-di­ethyl-9*H*-­fluorene with *N*-methyl­morpholine *N*-oxide (Seidel *et al.*, 2019 ▸). Single crystals of (**1**) were achieved *via* crystallization from a mixture of di­chloro­methane and *n*-hexane (1:1 *v*/*v*).

## Refinement   

Crystal data, data collection and structure refinement details are summarized in Table 2 ▸. The non-hydrogen atoms were refined anisotropically. All hydrogen atoms were positioned geometrically and allowed to ride on their parent atoms: C—H = 0.95 Å for aryl-H atoms, C—H = 0.99 Å for methyl­ene groups and C—H = 0.98 Å for methyl groups with *U*_iso_(H) = 1.5*U*_eq_(C) for methyl groups and *U*_iso_(H) = 1.2*U*_eq_(C) for other hydrogen atoms. The crystal structure of (**1**) was refined as a two-component twin with an approximate occupancy ratio of 63:37.

## Supplementary Material

Crystal structure: contains datablock(s) I. DOI: 10.1107/S2056989021009464/zq2265sup1.cif

Structure factors: contains datablock(s) I. DOI: 10.1107/S2056989021009464/zq2265Isup2.hkl

Supporting information file. DOI: 10.1107/S2056989021009464/zq2265Isup3.cml

CCDC reference: 2109160

Additional supporting information:  crystallographic information; 3D view; checkCIF report

## Figures and Tables

**Figure 6:**
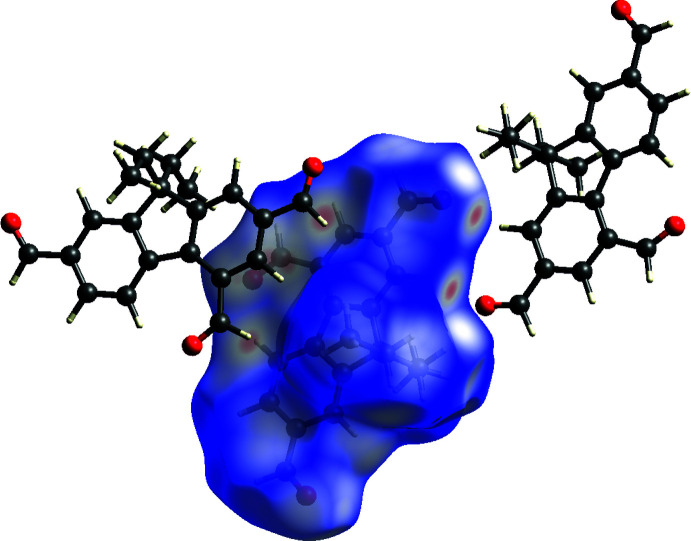
Hirshfeld surface for (**1**) mapped with *d*_norm_ and shape-index function showing neigbouring mol­ecules and the corresponding contacts.

**Table 1:** Hydrogen-bond geometry (Å, °)

*D*—H⋯*A*	*D*—H	H⋯*A*	*D*⋯*A*	*D*—H⋯*A*
C1—H1⋯O1^i^	0.95	2.59	3.512 (4)	165
C5—H5⋯O2	0.95	2.18	2.961 (4)	138
C5—H5⋯O3^ii^	0.95	2.67	3.350 (4)	129
C16—H16⋯O2^iii^	0.95	2.53	3.321 (4)	141
C17—H17*A*⋯O1^i^	0.99	2.68	3.611 (4)	157

Symmetry codes: (i) -x+1, -y+1, -z+1; (ii) -x, y-{\script{1\over 2}}, -z-{\script{1\over 2}}; (iii) -x, y+{\script{1\over 2}}, -z-{\script{1\over 2}}.

**Table 2:** Experimental details

Crystal data	
Chemical formula	C_20_H_18_O_3_
*M*_r_	306.34
Crystal system, space group	Monoclinic, *P*2_1_/*c*
Temperature (K)	150
*a*, *b*, *c* (Å)	15.6595 (9), 13.1466 (14), 7.6834 (15)
β (°)	93.146 (9)
*V* (Å^3^)	1579.4 (4)
*Z*	4
Radiation type	Mo *K*α
μ (mm^−1^)	0.09
Crystal size (mm)	0.38 × 0.30 × 0.15

Data collection	
Diffractometer	Stoe IPDS 2T
Absorption correction	–
No. of measured, independent and observed [*I* > 2σ(*I*)] reflections	13951, 13951, 8830
*R*_int_	?
(sin θ/λ)_max_ (Å^−1^)	0.594

Refinement	
*R*[*F*^2^ > 2σ(*F* ^2^)], *wR*(*F* ^2^), *S*	0.047, 0.127, 0.93
No. of reflections	13951
No. of parameters	211
H-atom treatment	H-atom parameters constrained
Δρ_max_, Δρ_min_ (e Å^−3^)	0.26, −0.24

Computer programs: *X-AREA* and *X-RED* (Stoe, 2009 ▸), *SHELXT2018/2* (Sheldrick, 2015*a* ▸), *SHELXL2018/3* (Sheldrick, 2015*b* ▸), *XP* (Sheldrick, 2008 ▸), *WinGX* (Farrugia, 2012 ▸), *publCIF* (Westrip, 2010 ▸) and *shelXle* (Hübschle *et al.*, 2011 ▸).

## supplementary crystallographic information

### Crystal data

**Table:**

C_20_H_18_O_3_	*F*(000) = 648
*M**_r_* = 306.34	*D*_x_ = 1.288 Mg m^−^^3^
Monoclinic, *P*2_1_/*c*	Mo *K*α radiation, λ = 0.71073 Å
*a* = 15.6595 (9) Å	Cell parameters from 6293 reflections
*b* = 13.1466 (14) Å	θ = 2.9–28.3°
*c* = 7.6834 (15) Å	µ = 0.09 mm^−^^1^
β = 93.146 (9)°	*T* = 150 K
*V* = 1579.4 (4) Å^3^	Piece, colorless
*Z* = 4	0.38 × 0.30 × 0.15 mm

### Data collection

**Table:**

Stoe IPDS 2T diffractometer	13951 independent reflections
Radiation source: sealed X-ray tube, 12 x 0.4 mm long-fine focus	8830 reflections with *I* > 2σ(*I*)
Plane graphite monochromator	θ_max_ = 25.0°, θ_min_ = 3.0°
Detector resolution: 6.67 pixels mm^-1^	*h* = −17→18
rotation method scans	*k* = −15→15
13951 measured reflections	*l* = −9→9

### Refinement

**Table:**

Refinement on *F*^2^	0 restraints
Least-squares matrix: full	Hydrogen site location: inferred from neighbouring sites
*R*[*F*^2^ > 2σ(*F*^2^)] = 0.047	H-atom parameters constrained
*wR*(*F*^2^) = 0.127	*w* = 1/[σ^2^(*F*_o_^2^) + (0.0713*P*)^2^] where *P* = (*F*_o_^2^ + 2*F*_c_^2^)/3
*S* = 0.93	(Δ/σ)_max_ < 0.001
13951 reflections	Δρ_max_ = 0.26 e Å^−^^3^
211 parameters	Δρ_min_ = −0.24 e Å^−^^3^

### Special details

**Table:**

Geometry. All esds (except the esd in the dihedral angle between two l.s. planes) are estimated using the full covariance matrix. The cell esds are taken into account individually in the estimation of esds in distances, angles and torsion angles; correlations between esds in cell parameters are only used when they are defined by crystal symmetry. An approximate (isotropic) treatment of cell esds is used for estimating esds involving l.s. planes.
Refinement. Refined as a two-component twin.

### Fractional atomic coordinates and isotropic or equivalent isotropic displacement parameters (Å^2^)

**Table:**

	*x*	*y*	*z*	*U*_iso_*/*U*_eq_	
O1	0.51535 (14)	0.34892 (18)	0.5435 (3)	0.0532 (7)	
O2	0.15641 (16)	0.18707 (17)	−0.0059 (3)	0.0604 (7)	
O3	0.03712 (16)	0.7414 (2)	−0.2592 (4)	0.0713 (8)	
C1	0.37790 (18)	0.4293 (2)	0.3116 (3)	0.0322 (7)	
H1	0.414572	0.480263	0.361567	0.039*	
C2	0.39372 (18)	0.3258 (2)	0.3466 (3)	0.0316 (7)	
C3	0.33905 (17)	0.2531 (2)	0.2722 (4)	0.0330 (6)	
H3	0.350875	0.183387	0.295850	0.040*	
C4	0.26770 (17)	0.2774 (2)	0.1647 (3)	0.0313 (7)	
C5	0.11308 (18)	0.4025 (2)	−0.0802 (4)	0.0372 (7)	
H5	0.099564	0.332138	−0.087467	0.045*	
C6	0.06274 (19)	0.4738 (2)	−0.1701 (4)	0.0412 (8)	
H6	0.014947	0.451369	−0.241394	0.049*	
C7	0.08030 (19)	0.5775 (2)	−0.1586 (4)	0.0385 (7)	
C8	0.15063 (18)	0.6120 (2)	−0.0543 (4)	0.0364 (7)	
H8	0.162721	0.682613	−0.044365	0.044*	
C9	0.28194 (18)	0.5627 (2)	0.1500 (4)	0.0313 (7)	
C10	0.30798 (17)	0.4554 (2)	0.2032 (3)	0.0294 (6)	
C11	0.25144 (17)	0.3818 (2)	0.1286 (3)	0.0291 (6)	
C12	0.18436 (17)	0.4364 (2)	0.0215 (3)	0.0305 (6)	
C13	0.20206 (18)	0.5414 (2)	0.0338 (3)	0.0311 (7)	
C14	0.46610 (18)	0.2932 (2)	0.4635 (4)	0.0379 (7)	
H14	0.474848	0.222039	0.477297	0.045*	
C15	0.2180 (2)	0.1882 (2)	0.0975 (4)	0.0436 (8)	
H15	0.236754	0.123771	0.140831	0.052*	
C16	0.0245 (2)	0.6512 (3)	−0.2560 (5)	0.0535 (9)	
H16	−0.024467	0.625546	−0.320103	0.064*	
C17	0.26251 (19)	0.6290 (2)	0.3086 (4)	0.0366 (7)	
H17A	0.315855	0.636730	0.382697	0.044*	
H17B	0.245393	0.697499	0.266210	0.044*	
C18	0.1931 (2)	0.5885 (2)	0.4212 (4)	0.0466 (8)	
H18A	0.190690	0.630388	0.526375	0.070*	
H18B	0.206083	0.518019	0.454380	0.070*	
H18C	0.137719	0.591187	0.355334	0.070*	
C19	0.35203 (19)	0.6155 (2)	0.0494 (4)	0.0388 (7)	
H19A	0.331460	0.684240	0.015221	0.047*	
H19B	0.403150	0.624283	0.129659	0.047*	
C20	0.3791 (2)	0.5611 (3)	−0.1127 (4)	0.0516 (9)	
H20A	0.419668	0.603613	−0.172536	0.077*	
H20B	0.328650	0.548144	−0.190883	0.077*	
H20C	0.406310	0.496296	−0.079554	0.077*	

### Atomic displacement parameters (Å^2^)

**Table:**

	*U*^11^	*U*^22^	*U*^33^	*U*^12^	*U*^13^	*U*^23^
O1	0.0435 (13)	0.0551 (15)	0.0591 (14)	−0.0035 (12)	−0.0151 (13)	0.0044 (13)
O2	0.0656 (15)	0.0508 (15)	0.0633 (15)	−0.0157 (12)	−0.0094 (14)	−0.0051 (12)
O3	0.0680 (16)	0.0551 (16)	0.0880 (18)	0.0021 (15)	−0.0219 (16)	0.0182 (15)
C1	0.0309 (15)	0.0348 (16)	0.0311 (13)	−0.0035 (13)	0.0026 (13)	−0.0008 (12)
C2	0.0302 (15)	0.0353 (16)	0.0296 (15)	0.0015 (13)	0.0045 (13)	0.0014 (12)
C3	0.0389 (15)	0.0300 (15)	0.0308 (14)	0.0026 (14)	0.0072 (14)	0.0007 (12)
C4	0.0349 (15)	0.0303 (16)	0.0292 (14)	−0.0029 (13)	0.0071 (14)	−0.0032 (12)
C5	0.0341 (16)	0.0363 (16)	0.0409 (16)	−0.0056 (14)	0.0000 (14)	−0.0041 (14)
C6	0.0320 (17)	0.049 (2)	0.0421 (17)	−0.0048 (15)	−0.0037 (15)	−0.0012 (14)
C7	0.0332 (16)	0.0450 (19)	0.0371 (15)	0.0006 (14)	−0.0003 (14)	0.0057 (14)
C8	0.0360 (16)	0.0350 (16)	0.0381 (16)	−0.0038 (14)	0.0003 (14)	0.0044 (13)
C9	0.0330 (15)	0.0263 (14)	0.0344 (14)	−0.0022 (12)	−0.0008 (13)	0.0009 (12)
C10	0.0303 (15)	0.0301 (15)	0.0280 (13)	−0.0010 (13)	0.0034 (13)	−0.0004 (12)
C11	0.0314 (15)	0.0289 (15)	0.0274 (13)	−0.0020 (13)	0.0042 (12)	−0.0018 (12)
C12	0.0287 (15)	0.0321 (15)	0.0309 (14)	−0.0024 (12)	0.0036 (13)	−0.0014 (13)
C13	0.0316 (15)	0.0328 (15)	0.0288 (13)	−0.0030 (13)	0.0001 (13)	−0.0002 (13)
C14	0.0355 (16)	0.0414 (17)	0.0371 (17)	0.0051 (15)	0.0056 (15)	0.0057 (15)
C15	0.050 (2)	0.0422 (19)	0.0389 (17)	−0.0085 (16)	0.0045 (17)	−0.0019 (15)
C16	0.044 (2)	0.055 (2)	0.060 (2)	−0.0005 (18)	−0.0105 (18)	0.0115 (19)
C17	0.0403 (17)	0.0308 (15)	0.0378 (15)	0.0010 (14)	−0.0054 (14)	−0.0035 (13)
C18	0.0519 (19)	0.0454 (19)	0.0427 (17)	0.0051 (16)	0.0043 (16)	−0.0022 (15)
C19	0.0378 (16)	0.0349 (16)	0.0430 (16)	−0.0097 (13)	−0.0046 (15)	0.0071 (14)
C20	0.0473 (19)	0.060 (2)	0.0478 (18)	−0.0153 (18)	0.0095 (16)	0.0028 (17)

### Geometric parameters (Å, º)

**Table:**

O1—C14	1.207 (3)	C9—C13	1.523 (4)
O2—C15	1.216 (3)	C9—C19	1.542 (4)
O3—C16	1.203 (4)	C9—C17	1.542 (4)
C1—C10	1.382 (4)	C10—C11	1.411 (4)
C1—C2	1.406 (4)	C11—C12	1.483 (4)
C1—H1	0.9500	C12—C13	1.410 (4)
C2—C3	1.386 (4)	C14—H14	0.9500
C2—C14	1.471 (4)	C15—H15	0.9500
C3—C4	1.390 (4)	C16—H16	0.9500
C3—H3	0.9500	C17—C18	1.522 (5)
C4—C11	1.421 (4)	C17—H17A	0.9900
C4—C15	1.484 (4)	C17—H17B	0.9900
C5—C6	1.384 (4)	C18—H18A	0.9800
C5—C12	1.400 (4)	C18—H18B	0.9800
C5—H5	0.9500	C18—H18C	0.9800
C6—C7	1.393 (4)	C19—C20	1.517 (5)
C6—H6	0.9500	C19—H19A	0.9900
C7—C8	1.401 (4)	C19—H19B	0.9900
C7—C16	1.479 (4)	C20—H20A	0.9800
C8—C13	1.381 (4)	C20—H20B	0.9800
C8—H8	0.9500	C20—H20C	0.9800
C9—C10	1.518 (4)		
			
C10—C1—C2	118.8 (2)	C13—C12—C11	107.8 (2)
C10—C1—H1	120.6	C8—C13—C12	121.2 (2)
C2—C1—H1	120.6	C8—C13—C9	127.0 (2)
C3—C2—C1	119.4 (2)	C12—C13—C9	111.8 (2)
C3—C2—C14	119.2 (3)	O1—C14—C2	125.7 (3)
C1—C2—C14	121.4 (2)	O1—C14—H14	117.2
C2—C3—C4	123.0 (3)	C2—C14—H14	117.2
C2—C3—H3	118.5	O2—C15—C4	128.2 (3)
C4—C3—H3	118.5	O2—C15—H15	115.9
C3—C4—C11	117.9 (2)	C4—C15—H15	115.9
C3—C4—C15	114.4 (3)	O3—C16—C7	124.2 (3)
C11—C4—C15	127.7 (2)	O3—C16—H16	117.9
C6—C5—C12	118.5 (3)	C7—C16—H16	117.9
C6—C5—H5	120.7	C18—C17—C9	115.5 (2)
C12—C5—H5	120.7	C18—C17—H17A	108.4
C5—C6—C7	121.8 (2)	C9—C17—H17A	108.4
C5—C6—H6	119.1	C18—C17—H17B	108.4
C7—C6—H6	119.1	C9—C17—H17B	108.4
C6—C7—C8	119.9 (3)	H17A—C17—H17B	107.5
C6—C7—C16	120.0 (3)	C17—C18—H18A	109.5
C8—C7—C16	120.1 (3)	C17—C18—H18B	109.5
C13—C8—C7	118.8 (3)	H18A—C18—H18B	109.5
C13—C8—H8	120.6	C17—C18—H18C	109.5
C7—C8—H8	120.6	H18A—C18—H18C	109.5
C10—C9—C13	100.8 (2)	H18B—C18—H18C	109.5
C10—C9—C19	111.4 (2)	C20—C19—C9	116.0 (2)
C13—C9—C19	111.9 (2)	C20—C19—H19A	108.3
C10—C9—C17	112.0 (2)	C9—C19—H19A	108.3
C13—C9—C17	112.1 (2)	C20—C19—H19B	108.3
C19—C9—C17	108.6 (2)	C9—C19—H19B	108.3
C1—C10—C11	122.2 (2)	H19A—C19—H19B	107.4
C1—C10—C9	125.8 (2)	C19—C20—H20A	109.5
C11—C10—C9	112.0 (2)	C19—C20—H20B	109.5
C10—C11—C4	118.8 (2)	H20A—C20—H20B	109.5
C10—C11—C12	107.6 (2)	C19—C20—H20C	109.5
C4—C11—C12	133.6 (2)	H20A—C20—H20C	109.5
C5—C12—C13	119.8 (2)	H20B—C20—H20C	109.5
C5—C12—C11	132.4 (2)		
			
C10—C1—C2—C3	−0.1 (4)	C10—C11—C12—C5	180.0 (3)
C10—C1—C2—C14	−178.8 (3)	C4—C11—C12—C5	0.6 (6)
C1—C2—C3—C4	−0.7 (4)	C10—C11—C12—C13	0.1 (3)
C14—C2—C3—C4	178.0 (3)	C4—C11—C12—C13	−179.3 (3)
C2—C3—C4—C11	0.8 (4)	C7—C8—C13—C12	−0.6 (5)
C2—C3—C4—C15	179.6 (3)	C7—C8—C13—C9	178.7 (3)
C12—C5—C6—C7	−1.3 (5)	C5—C12—C13—C8	−0.6 (4)
C5—C6—C7—C8	0.1 (5)	C11—C12—C13—C8	179.3 (3)
C5—C6—C7—C16	−179.6 (3)	C5—C12—C13—C9	−180.0 (3)
C6—C7—C8—C13	0.9 (5)	C11—C12—C13—C9	−0.1 (3)
C16—C7—C8—C13	−179.4 (3)	C10—C9—C13—C8	−179.4 (3)
C2—C1—C10—C11	0.9 (4)	C19—C9—C13—C8	−60.9 (4)
C2—C1—C10—C9	−179.3 (3)	C17—C9—C13—C8	61.4 (4)
C13—C9—C10—C1	−179.8 (3)	C10—C9—C13—C12	0.0 (3)
C19—C9—C10—C1	61.4 (3)	C19—C9—C13—C12	118.5 (3)
C17—C9—C10—C1	−60.5 (4)	C17—C9—C13—C12	−119.2 (3)
C13—C9—C10—C11	0.0 (3)	C3—C2—C14—O1	−175.8 (3)
C19—C9—C10—C11	−118.8 (2)	C1—C2—C14—O1	2.9 (5)
C17—C9—C10—C11	119.3 (3)	C3—C4—C15—O2	−175.4 (3)
C1—C10—C11—C4	−0.8 (4)	C11—C4—C15—O2	3.2 (5)
C9—C10—C11—C4	179.4 (3)	C6—C7—C16—O3	−176.7 (4)
C1—C10—C11—C12	179.7 (3)	C8—C7—C16—O3	3.5 (6)
C9—C10—C11—C12	−0.1 (3)	C10—C9—C17—C18	−57.6 (3)
C3—C4—C11—C10	−0.1 (4)	C13—C9—C17—C18	54.8 (3)
C15—C4—C11—C10	−178.6 (3)	C19—C9—C17—C18	179.0 (2)
C3—C4—C11—C12	179.2 (3)	C10—C9—C19—C20	56.6 (3)
C15—C4—C11—C12	0.7 (5)	C13—C9—C19—C20	−55.3 (3)
C6—C5—C12—C13	1.5 (4)	C17—C9—C19—C20	−179.6 (2)
C6—C5—C12—C11	−178.3 (3)		

### Hydrogen-bond geometry (Å, º)

**Table:**

*D*—H···*A*	*D*—H	H···*A*	*D*···*A*	*D*—H···*A*
C1—H1···O1^i^	0.95	2.59	3.512 (4)	165
C5—H5···O2	0.95	2.18	2.961 (4)	138
C5—H5···O3^ii^	0.95	2.67	3.350 (4)	129
C16—H16···O2^iii^	0.95	2.53	3.321 (4)	141
C17—H17*A*···O1^i^	0.99	2.68	3.611 (4)	157

Symmetry codes: (i) −*x*+1, −*y*+1, −*z*+1; (ii) −*x*, *y*−1/2, −*z*−1/2; (iii) −*x*, *y*+1/2, −*z*−1/2.
